# Supplementary figures and images for: Characterization of the Chloroplast Genome of Trentepohlia odorata (Trentepohliales, Chlorophyta), and Discussion of its Taxonomy
Source: Int J Mol Sci. 2019 Apr 10;20(7):1774. doi: 10.3390/ijms20071774 (PMC6480257; doi:10.3390/ijms20071774)

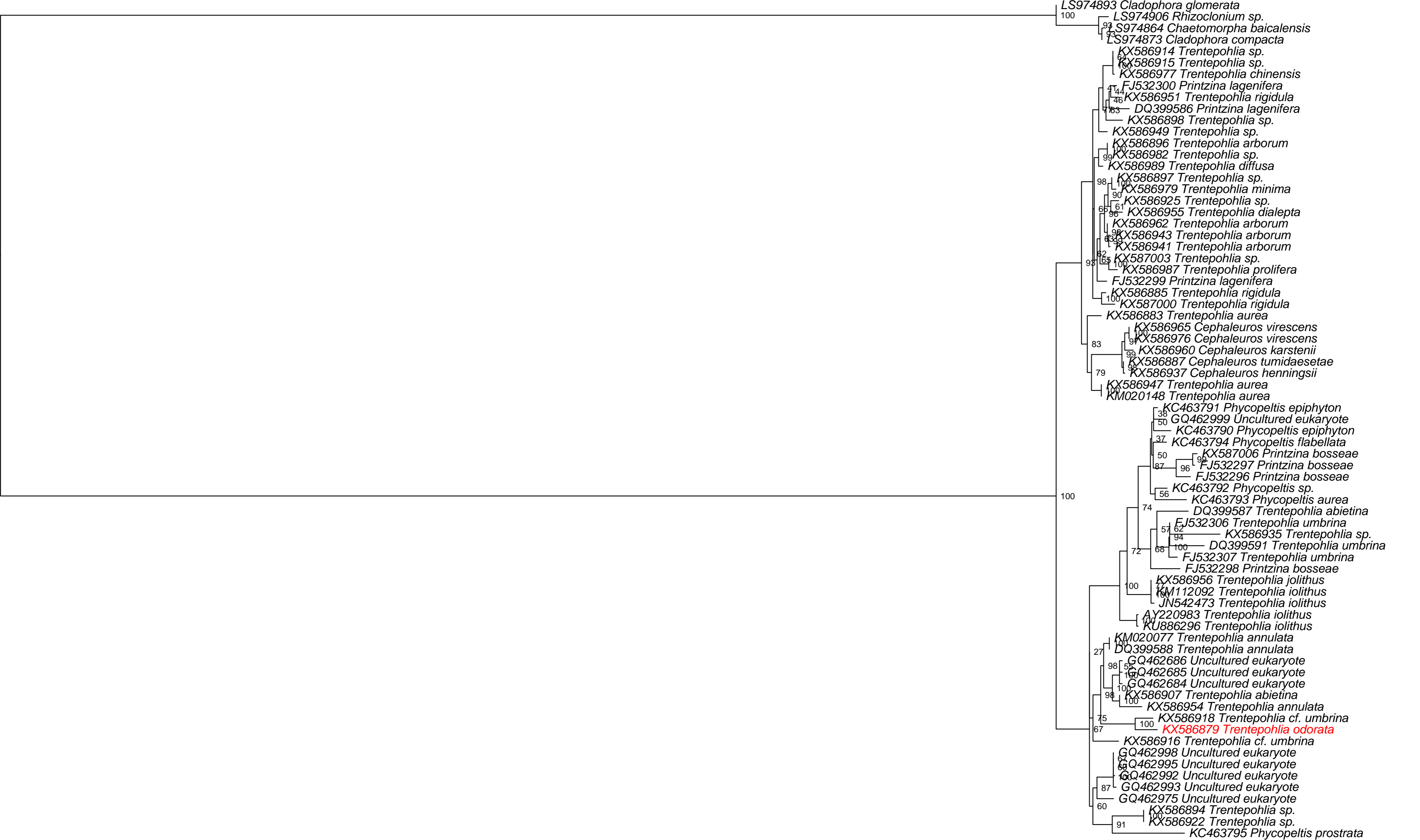

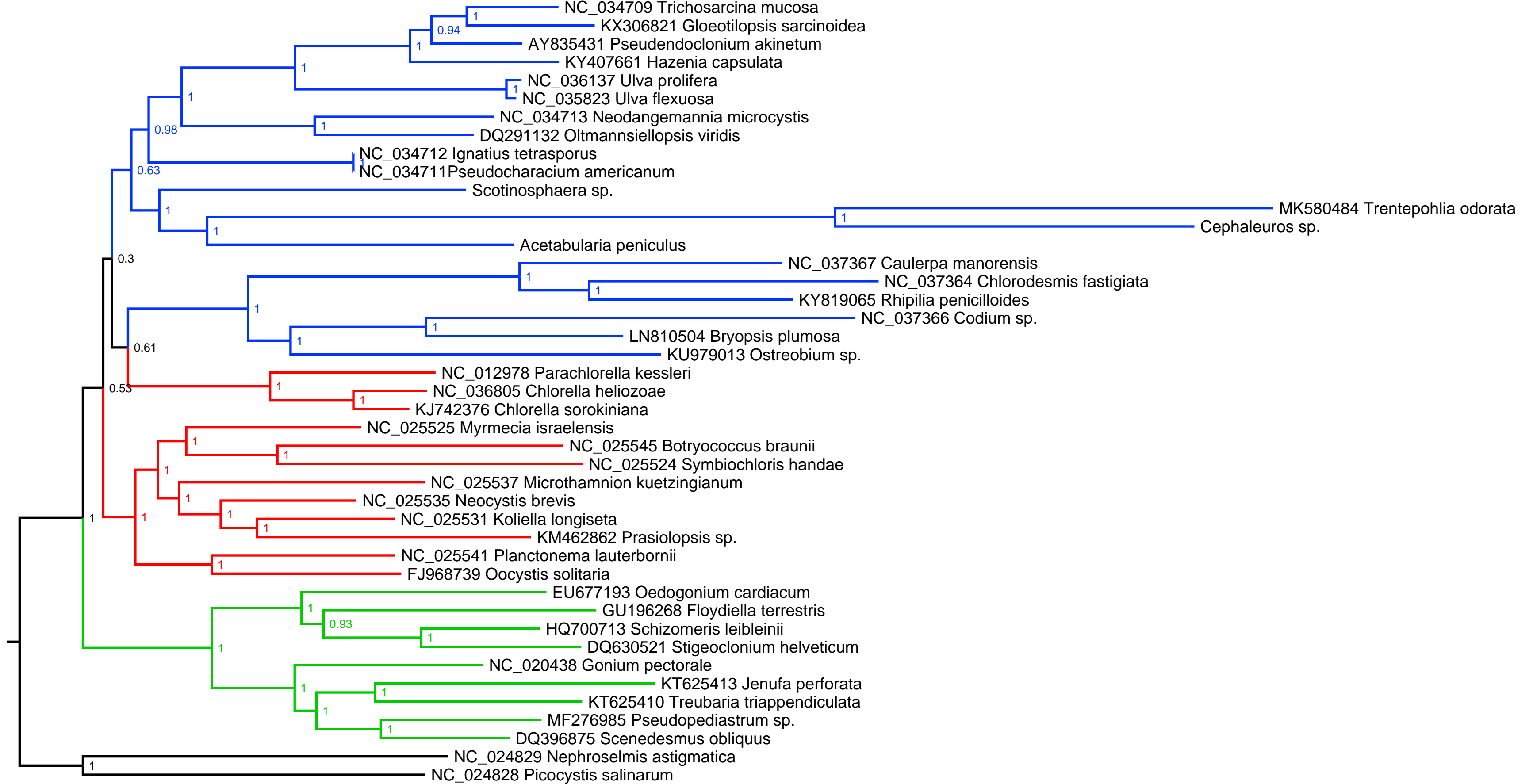

## Multi FASTA (all. fa)

Trentepohlia\_sp.\_JZ1317

Supplement: Supplementary file 1 [file ijms-20-01774-s001.pdf]
